# Validation of Four Prediction Scores for Cardiac Surgery-Associated Acute Kidney Injury in Chinese Patients

**DOI:** 10.21470/1678-9741-2017-0116

**Published:** 2017

**Authors:** Wuhua Jiang, Jiarui Xu, Bo Shen, Chunsheng Wang, Jie Teng, Xiaoqiang Ding

**Affiliations:** 1 Department of Nephrology of Zhongshan Hospital of Fudan University Shanghai Medical College, Shanghai, China.; 2 Shanghai Kidney and Dialysis Institute of Fudan University Shanghai Medical College, Shanghai, China.; 3 Department of Cardiovascular Surgery of Zhongshan Hospital of Fudan University Shanghai Medical College, Shanghai, China.; 4 Shanghai Kidney and Blood Purification Laboratory of Zhongshan Hospital of Fudan University Shanghai Medical College, Shanghai, China.

**Keywords:** Acute Kidney Injury, Cardiac Surgical Procedures, Risk Assessment, Renal Replacement Therapy, Validation Studies

## Abstract

**Objective:**

To assess the clinical value of four models for the prediction of cardiac surgery-associated acute kidney injury (CSA-AKI) and severe AKI which renal replacement therapy was needed (RRT-AKI) in Chinese patients.

**Methods:**

1587 patients who underwent cardiac surgery in the department of cardiac surgery in the Zhongshan Hospital, Fudan University, between January 2013 and December 2013 were enrolled in this research. Evaluating the predicting value for cardiac surgery-associated AKI (AKICS score) and RRT-AKI (Cleveland score, SRI and Mehta score) by Hosmer-Lemeshow goodness-of-fit test for the calibration and area under receiver operating characteristic curve (AUROC) for the discrimination.

**Results:**

Based on 2012 KDIGO (Kidney Disease: Improving Global Outcomes) AKI definition, the incidence of AKI and RRT-AKI was 37.4% (594/1587) and 1.1% (18/1587), respectively. The mortality of AKI and RRT-AKI was 6.1% (36/594) and 66.7% (12/18), respectively, while the total mortality was 2.8% (44/1587). The discrimination (AUROC=0.610) for the prediction of CSA-AKI of AKICS was low, while the calibration (x^2^=7.55, *P*=0.109) was fair. For the prediction of RRT-AKI, the discrimination of Cleveland score (AUROC=0.684), Mehta score (AUROC=0.708) and SRI (AUROC=0.622) were not good; while the calibration of them were fair (Cleveland score x^2^=1.918, *P*=0.166; Mehta score x^2^=9.209, *P*=0.238; SRI x^2^=2.976, *P*=0.271).

**Conclusion:**

In our single-center study, based upon valve surgery dominant and less diabetes mellitus patients, according to KDIGO AKI definition, the predictive value of the four models, combining discrimination and calibration, for respective primary event, were not convincible.

**Table:** 

Abbreviations, acronyms & symbols	
AKI	= Acute kidney injury
AUROC	= Area under receiver operating characteristics curve
CABG	= Coronary artery bypass grafting
CPB	= Cardiopulmonary bypass
CSA-AKI	= Cardiac surgery-associated acute kidney injury
KDIGO	= Kidney Disease: Improving Global Outcomes
RRT-AKI	= Renal replacement therapy-acute kidney injury
SCr	= Serum creatinine
SD	= Standard deviation

## INTRODUCTION

With the technique of cardiac surgery booming in the big developing countries like China, Brazil and India, more and more complex procedures can be performed. Meanwhile, the following complications of surgery may emerge as well, which will be a huge burden of national health cost^[1]^. Acute kidney injury (AKI) is one of these common complications after cardiac surgery with reported incidences over 30% while mortality increased fourfold and even slight renal function changes were reported to influence short- and long-term survival rates after cardiac surgery^[2,3]^. Since early identifying the patients who are at high risk of developing into cardiac surgery associated acute kidney injury (CSA-AKI) may improve prognosis, in the last two decades various predictive models have been developed to forecast CSA-AKI or renal replacement therapy (RRT)-AKI required after cardiac surgery. Among them, adequate predictive value has been validated in the Caucasians cohorts^[4,5]^ in four risk scores^[6-9]^. However, with the new 2012 Kidney Disease: Improving Global Outcomes (KDIGO) AKI definition being popularized^[10]^, the predictive value for predicting CSA-AKI, RRT-AKI become doubtful due to the updated definition, with which some "subclinical AKI" can be defined.

The aim of this study is to validate the predictive value of four predictive scores for their respective outcome in Chinese patients, in which cohort valve surgeries are dominant, and less diabetes^[11]^.

## METHODS

### Patients

In our retrospective study, we included patients at department of cardiac surgery between January 2013 and December 2013, and extracted data from a computerized cardiac surgical database. Patients (aged > 18 years) who underwent cardiac surgical procedures [coronary artery bypass grafting (CABG) alone, mitral or aortic valve surgery alone, or combination of CABG and aortic or mitral valve surgery] with cardiopulmonary bypass (CPB) were enrolled. In addition, according to common exclusion criteria derived from these four scores, patients who were on RRT preoperatively, those who underwent heart transplant or assist device insertion and patients who denied access to their medical records for the purpose of research were also excluded. In order to evaluate predictive value of different models, the prediction scores were calculated and summed as in the original studies^[6-9]^.

Patients who did not meet the original inclusion criteria for a certain model were excluded from the analysis of that specific model. If there were more than one cardiac surgery procedures performed during the same hospitalization, only the data with the first surgery were considered. All the patients included were followed until discharge or death. The study was approved by the ethical committee of the Zhongshan Hospital and all participants provided written informed consent before inclusion.

### Outcomes

The four prediction scores analyzed in this study were Cleveland score, Mehta score, SRI and AKICS score. The brief description and characteristics of the scores were shown in Table 1.

**Table 1:** Predictors in each score.

Prediction	Cleveland Score		Mehta Score		SRI Score		AKICS Score	
	RRT-AKI		RRT-AKI		RRT-AKI		AKI	
Variable	Definition	Score	Definition	Score	Definition	Score	Definition	Score
Age			Varies	Varies			> 65	2.3
Race			Non-white	2				
Gender	Female	1						
Preoperative kidney function	SCr 1.2-2.1 mg/dL	2	SCr	Varies	GFR, 31-60 mL/min	1	Preoperative SCr > 1.2 mg/dL	3.1
	SCr > 2.1 mg/dL	5			GFR ≤ 30 mL/min	2		
CHF	Yes	1						
NYHA Class					4	3	3 or 4	3.2
Diabetes							Preoperative capillary glucose > 140 mg/dL	1.7
COPD	Yes	1	Yes	3				
Recent MI (< 21 d)			Yes	3				
LVEF	< 35%	1			≤ 40%	1		
Previous surgery	Yes	1	Yes	3	Yes	1		
Preoperative IABP	Yes	2			Yes	1		
Cardiogenic shock			Yes	7				
Timing of surgery	Emergence	2			Non-selective	1		
CPB time							> 120 min	1.8
Postoperative CVP							> 14 cmH2O	1.7
LCOS							Yes	2.5
Type of surgery	CABG only	0	CABG only	0	Other than CABG	1	Combined surgery	3.7
	Valve only	1	Aortic valve only	2				
	CABG + valve	2	Aortic valve + CABG	5				
			Mitral valve only	4				
			Mitral valve + CABG	7				
Score range		0-17		0-83		0-8		0-20

AKI=acute kidney injury; RRT-AKI=AKI which renal replacement therapy is needed; CABG=coronary artery bypass grafting; CHF=congestive heart failure; COPD=chronic obstructive pulmonary disease; CPB=cardiopulmonary bypass; CVD=cerebral vascular disease; CVP=central venous pressure by ICU admittance; DM=diabetes mellitus; eGFR=estimated glomerular filtration rate, as calculated based on the Cockcroft-Gault formulae for SRI score validation; GFR=glomerular filtration rate; IABP=intra-aortic balloon pump; LCOS=low cardiac output syndrome; LVEF=left ventricular ejection fraction; MI=myocardial infarction; NYHA=New York Heart Association; SCr=serum creatinine; SD=standard deviation

RRT-AKI, defined with the initiation of dialysis in the postoperative course until the discharge, was the outcome for validation of Cleveland score, Mehta score and SRI. The dialysis was initiated at the consulting nephrologists based on the indication including uremia, acidosis, hyperkalemia or severe fluid overload. CSA-AKI defined with KDIGO guideline was the outcome for validation of AKICS score.

KDIGO AKI definition:

- Increase in serum creatinine (SCr) by ≥ 0.3 mg/dL (≥ 26.5 µmol/L) within 48 hours; or
- Increase in SCr to ≥ 1.5 times baseline, which is known or presumed to have occurred within the prior 7 days; or
- Urine volume < 0.5 mL/kg/h for 6 hours.

### Statistical Analysis

Statistical analysis was carried out by SPSS statistics for Windows (Version 20.0. Armonk, NY, USA, IBM Corp). Continuous variables were expressed as mean ± standard deviation (SD), and analyzed by unpaired t-tests, with Welch's adjustment when necessary. Non-parametric variables were expressed as median and 25-75 percentiles and analyzed by Mann-Whitney U test.

Categorical variables were expressed as absolute (n) and relative (%) frequency, and were analyzed by Pearson's 2-test or Fisher's exact test, whenever appropriate. Significant level was considered with *P*<0.05. Area under receiver operating characteristic curves (AUROC) and Hosmer-Lemeshow goodness-of-fit test were utilized to evaluate discrimination and calibration of every predictive risk score. Statistically, calibration refers to the agreement between observed and predicted risk and Hosmer-Lemeshow *P* value > 0.05 means good calibration. Discrimination refers to the capacity of separating people with disease from people without disease and the AUROC > 0.80 indicates good discrimination.

## RESULTS

A total of 1609 patients were included in our study. After excluding cases with preoperative RRT (n=4), left ventricular device (n=2), missing data (n=11) and intra-operative and early-post-operative death (< 24h) (n=5), 1587 patients were included to the validation (Table 2). Due to the original exclusion criteria, the cases with emergency surgeries (n=2) were not qualified into the validation of AKICS score.

**Table 2:** Characteristics of the patients in the validation cohort.

Preoperative	Non-AKI (N=993)	AKI (n=594)	*P*
Male	542 (54.6%)	434 (73.1%)	< 0.01
Age, mean (SD), years	56.8 (12.3)	58.7 (11.7)	0.08
Kidney function			
Serum creatinine, mean (SD) mg/dL	0.83 (0.2)	1.04 (0.40)	< 0.01
eGFR, mean (SD) mL/min/1.73 m^2^	109.4 (30.4)	85.9 (25.2)	< 0.01
Comorbidities			
Hypertension	320 (32.2%)	204 (34.3%)	0.408
DM	136 (13.7%)	60 (10.1%)	0.04
COPD	0	0	
CVD	3 (0.3%)	4 (0.7%)	0.43
Cardiac function			
NYHA classification > 2	541 (54.5%)	363 (61.1%)	< 0.01
LVEF ≤ 35%	101 (10.2%)	147 (24.4%)	< 0.01
Previous cardiac surgery	9 (0.9%)	8 (1.3%)	0.454
Intraoperative			
Emergency	2 (0.2%)	0	0.531
Procedure			
Valve	605 (60.9%)	428 (72.1%)	< 0.01
CABG	342 (34.4%)	133 (22.4%)	< 0.01
Valve + CABG	46 (4.6%)	33 (5.6%)	< 0.01
CPB time (min)	89 (71,113)	100 (79,124)	< 0.01
Postoperative			
LCOS	3 (0.3%)	13 (2.2%)	< 0.01
CVP, mean (SD), cmH2O	8.4 (2.7)	10.9 (2.9)	< 0.01
Prognosis			
28-day mortality	8 (0.8%)	36 (6.1%)	< 0.01

AKI=acute kidney injury; RRT-AKI=AKI which renal replacement therapy is needed; CABG=coronary artery bypass grafting; COPD=chronic obstructive pulmonary disease; CPB=cardiopulmonary bypass; CVD=cerebral vascular disease; CVP=central venous pressure by ICU admittance; DM=diabetes mellitus; eGFR=estimated glomerular filtration rate, as calculated based on the Cockcroft- Gault formulae for SRI score validation; LCOS=low cardiac output syndrome; LVEF=left ventricular ejection fraction; NYHA=New York Heart Association; SCr=serum creatinine; SD=standard deviation

**Fig. 1:**
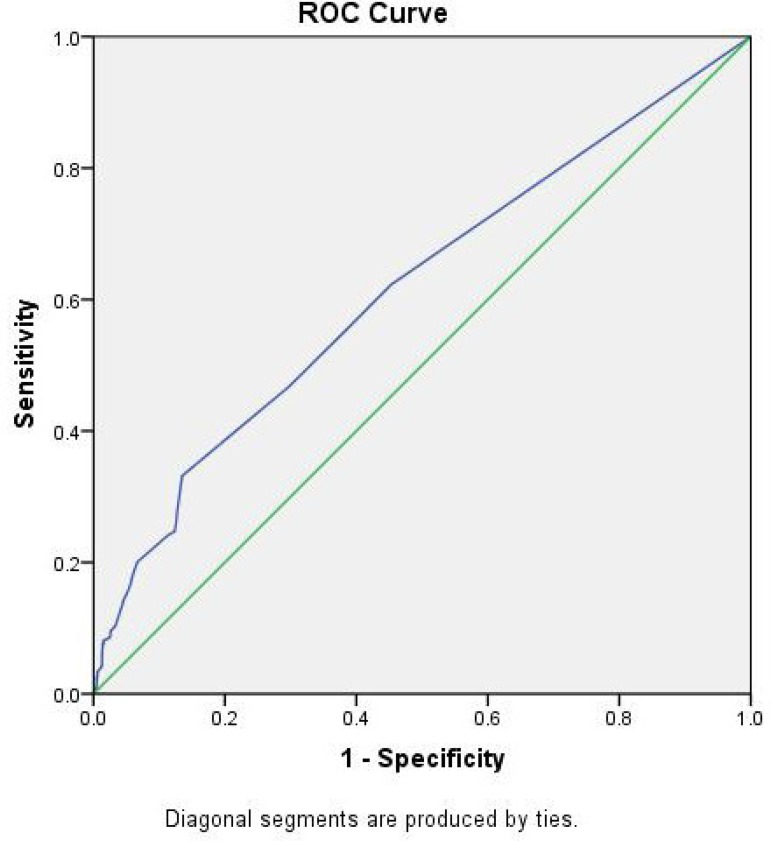
ROC curve for the prediction of CSA-AKI of AKICS score.

**Fig. 2:**
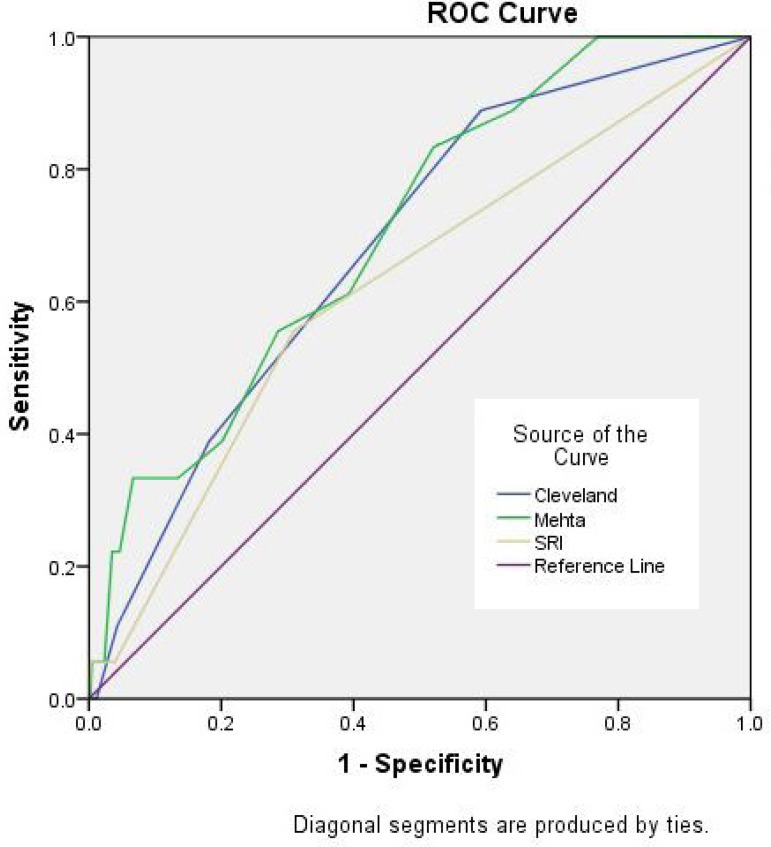
ROC curves for the prediction of RRT-AKI of Cleveland score, Mehta score and SRI.

The incidence of AKI was 37.4% (594/1587), while the incidence of RRT-AKI was 1.1% (18/1587) and the mortality of AKI and RRT-AKI were 6.1% (36/594) and 66.7% (12/18), respectively. The total mortality was 2.8% (44/1587). The discrimination (AUC, 95% CI) for both the prediction of CSA-AKI of AKICS (0.610, 95% CI 0.59 to 0.65) (Figure 1) and the prediction of RRT-AKI of Cleveland score (0.684, 95% CI 0.57 to 0.79) and SRI (0.622, 95% CI 0.49 to 0.75) were low, while Mehta score (0.708, 95% CI 0.60 to 0.81) indicated barely fair discrimination (Figure 2). On the other side, calibration of AKICS score (x^2^=7.55, *P*=0.109), Cleveland score (x^2^=1.918, *P*=0.166), Mehta score (x^2^=9.209, *P*=0.238) and SRI (x^2^=2.976, *P*=0.271) were fair. Compared to the expected incidence, in our study, AKICS score far underestimated the incidence of AKI, and the other models overestimated the incidence of RRT-AKI slightly (Table 3).

**Table 3:** Comparison between predicted and observed outcomes for each score.

	AKI		RRT-AKI	
	AKICS (n=1585)	Cleveland (n=1587)	Mehta (n=1587)	SRI (n=1587)
Predicted	14%	1.7%	1.4%	1.3-2.2%
Observed	37.4%	1.1%	1.1%	1.1%

AKI=acute kidney injury

The predicted incidences of each outcome were derived from the origin papers.

Although the purpose of the study was to evaluate patients according to the KDIGO criteria, we presented a subanalysis using the AKICS criteria for CSA-AKI.

CSA-AKI criteria in AKICS study: an increase of SCr levels above than 2.0 mg/dL in patients with baseline SCr lower than 1.5 mg/dL. In patients with baseline creatinine between 1.5 and 3.0 mg/dL, AKI was defined as a creatinine increase of 50% over the baseline value.

The incidence of CSA-AKI resulted from the AKICS criteria is lower than that from the KDIGO definition (22.8% *vs.* 37.4%, *P*<0.001).

## DISCUSSION

As one of the most common complications of cardiac surgery, AKI plays an important role in worsening prognosis. In the past decades, the incidence of CSA-AKI varies between 8.9% and 39%, according to different definition^[2,3]^. Some revealed reasons for the wide range of incidence are the difference between underlying disease, procedure types, comorbidities, ethics and medical care development in global population.

Since early prediction in those patients at high risk of developing CSA-AKI may contribute to prevention of complications, in the last two decades various predictive models have been developed to forecast CSA-AKI or RRT-AKI after cardiac surgery. Among those risk scores, adequate predictive power in the method published by Palomba et al.^[7]^, the Cleveland Clinic score^[8]^, the scoring system published by Mehta et al.^[6]^ and the simplified renal index score^[9]^ were validated among Caucasians^[4,5]^. However, with the developing of AKI academic community, especially the publication of KDIGO guideline and recognition of intraoperative and post-operative risk factors, utilizing these risk scores in population other than Caucasians, and cohorts with more valve surgeries and less diabetes become questionable. With the development of medical technology, the cardiac surgery in developing countries like China is booming. Meanwhile, the complication of surgery and economic burden are also increasing due to relatively low medical technology and management. Compared to the existing score derivation cohorts, the incidence of CSA-AKI is much higher in developing countries like Brazil and China^[11-14]^. Thus, it is important to identify whether or not these existing scores are good enough to predict respective outcome in patients in developing countries.

In 2007, Palomba et al.^[7]^ carried out a single-center study to generate a risk score to predict CSA-AKI with a cohort of 603 patients. In that study, the result of internal validation was good with AUROC 0.84, which meant a good predictive power of AKI after cardiac surgery. By that time, the authors had recognized the worse prognosis and long-term CKD evolvement could be resulted from even slightly AKI. They also focused on the intraoperative and early postoperative risk factors contributing to CSA-AKI. This has been proven a promising milestone for subsequent authors to build predictive risk scores. However, the validation in our study is not as good as that. Potential reason may include: (1) The definition of AKI in the primary study was an increase of SCr levels above than 2.0 mg/dL in patients with baseline SCr lower than 1.5 mg/dL. In patients with baseline creatinine between 1.5 and 3.0 mg/dL, AKI was defined as a creatinine increase of 50% over the baseline value. This urine output lacking definition may result in the missing diagnosis of CSA-AKI compared with the KDIGO definition. (2) The Asian population in the primary study is 3.8%, while we know the racial discrepancy may affect the validation result^[15]^.

As for the three risk scores predicting RRT-AKI, they were generated upon deliberate design and large sample cohorts, Mehta score^[6]^ and SRI score^[9]^ were established with multicenter studies, and their predictive power have been validated sufficient among Caucasian population^[4,5]^. However, their predictive power in our study were not good enough, and we tried to find the reason. Maybe the following can explain.

Firstly, the endpoint event of the Cleveland score, Mehta score and SRI is RRT-AKI. Though up till now, whether early RRT is beneficial remains debatable^[16]^, some studies have shown early initiation of RRT can reduce short-term mortality^[17]^. However, the variability of local practices in when to start RRT may affect the incidence significantly. As is known to all, the key problem of risk scores is their derivation, which is strongly dependent on characteristics of the derivation cohort and the statistical methods^[18]^, especially when the endpoint has a low incidence and is multifactorial. The RRT-AKI incidence in our cohort is 1.1%, slightly lower than that in those three scores (Cleveland 1.7%, Mehta 1.4% and SRI 1.3-2.2%). This may be one of the reasons that result in their insufficient predictive power in our study.

Secondly, despite of strict similar inclusion or exclusion criteria being utilized, there remained much difference among our cohort and the derivation cohorts. The proportion of valve surgery is much higher in developing countries like China than the previous risk scores developing centers, in which CABG is majority. Compared to CABG, the pathophysiology and pathological impairment are diversified. Patients who undergo valve surgery typically demonstrate low stroke volume related to regurgitation, which increases the vulnerability of the kidney to injury during cardiac surgery. The comorbidities in developing countries like Chinese patients are also different from Western population. In our derivation cohort, the ratio of hypertension and diabetes mellitus is also remarkable, lower than the previous develop population^[19]^.

Nonetheless, our validation did not make the scores useless. The predictors can be further analyzed with other newly found risk factors^[20]^ or biomarkers so as to establish new brand risk scores^[21]^. Along with the discovery of intraoperative and postoperative predictors, a dynamic predictive model for CSA-AKI/RRT-AKI based on the KDIGO AKI definition, which might be applied to predict the incidence of CSA-AKI from preoperative to early postoperative periods, shall meet the need. Meanwhile some limitation of our study must be noted, our validation is a single-center study, with relatively lower sample amount.

## CONCLUSION

We carried out this study to validate four risk scores predicting CSA-AKI or RRT-AKI. Although all scores presented good calibration in our cohort, their discrimination were barely satisfactory with an underestimated CSA-AKI incidence by AKICS score and overestimated RRT-AKI incidence by Cleveland score, Mehta score and SRI score. However, the risk factors in the scores can be further analyzed to generate reliable new risk scores.

**Table:** 

Authors' roles & responsibilities	
WJ	Substantial contributions to the conception or design of the work; acquisition, analysis, or interpretation of data for the work; drafting the work or revising it critically for important intellectual content; final approval of the version to be published
JX	Substantial contributions to the conception or design of the work; acquisition, analysis, or interpretation of data for the work; final approval of the version to be published
BS	Substantial contributions to the conception or design of the work; acquisition, analysis, or interpretation of data for the work; final approval of the version to be published
CW	Final approval of the version to be published
JT	Drafting the work or revising it critically for important intellectual content; final approval of the version to be published
XD	Agreement to be accountable for all aspects of the work in ensuring that questions related to the accuracy or integrity of any part of the work are appropriately investigated and resolved; final approval of the version to be published
